# Is the Simple Closure Technique Effective in the Treatment of Genital Fistulas?

**DOI:** 10.1155/2013/672540

**Published:** 2013-02-13

**Authors:** Eylem Unlubilgin, Tolgay Tuyan İlhan, Ahmet Akin Sivaslioglu, Ismail Dolen

**Affiliations:** ^1^Ankara Etlik Zubeyde Hanim Women's Health Teaching and Research Hospital, Yeni Etlik Cadde No. 55, Etlik, 06010 Ankara, Turkey; ^2^Van Özalp Devlet Hastanesi, Cumhuriyet Mahalle No.3 Özalp Merkez, Özalp, 65800 Van, Turkey; ^3^Ankara Ataturk Training and Research Hospital, Bilkent yolu 3. Km Çankaya, 06800 Ankara, Turkey

## Abstract

*Aim*. Genitourinary fistulas are bothersome clinical entities not only for the patient but also for the treating surgeon as well. A lot of surgical procedures have been proposed; however, most of the fistulas can be easily treated with plain surgical techniques, such as the simple surgical closure of the fistula tract. *Material and Method*. The study was carried out in the urogynecology department of Ankara Etlik Zübeyde Hanım Maternity Training and Research Hospital. The study included 12 cases with vesicovaginal fistulas and 15 cases with rectovaginal fistulas. Twenty-six patients underwent simple surgical closure technique. The age, the referral time to the hospital, the longest diameter of the fistula opening, the hospitalization time, the follow-up period and identifiable risk factors of the patients were evaluated. *Results*. Caeserean section was detected as primary risk factor for vesicovaginal fistulas and prolonged labor was detected as the most important risk factor for rectovaginal fistulas. In our study, we found that the simple closure technique cured 91% of vesicovaginal fistulas and 93% of rectovaginal fistulas. *Conclusion*. The simple closure technique has very high cure rates for both vesicovaginal and rectovaginal fistulas when the longest diameter of the fistula openings is ≤5 mm.

## 1. Introduction

Female genital fistulas lead to social and psychologic stress by deteriorating the quality of life because of bad smell due to continuous leakage of urine into the vagina or uncontrolled passage of flatus or stool through the vagina. These conditions are serious health problems and have a profound effect on the patient's emotional well-being [1, 2]. Genital fistulas could be classified as urogenital fistulas (UGF) and rectovaginal fistulas (RVF). UGFs may occur between the urinary bladder and the vagina (vesicovaginal fistula (VVF) the most common type); between the bladder and cervix or uterus; between the ureter and vagina, uterus or cervix; and between the urethra and vagina.

The predominant cause of VVF is prolonged obstructed labor in the developing world, whereas gynecological surgery (mainly hysterectomy) is the most seen risk factor in the developed world [2]. The most common etiology of RVF is obstetrical injury [3]. Other reasons include radiation, inflammatory bowel disease, pelvic operations, forceful coitus, and neoplasms. The exact incidence of genital fistulas which is directly related to the socioeconomic level of the affected population is unknown due to data inadequacy in the developing world [2–4]. Genital fistulas can be treated via abdominal or vaginal routes.

Moreover, the fistula surgery can be managed laparoscopically [5, 6].

In this study, we aimed to report the efficiency of the simple surgical closure technique in the treatment of the genital fistulas.

## 2. Materials and Methods

The study was carried out in the Urogynecology Department of Ankara Etlik Zübeyde Hanım Maternity Training and Research Hospital between the dates of January 01, 2003 and December 12, 2006. The ethics committee of the hospital accepted the study (the ethical committee's report number and the date is July/30 December 18, 2008) and also patients gave an informed consent to take part in this study. This study included 27 patients with a history of continuous leakage of urine or feces. The age, the referral time to the hospital, the longest diameter of the fistula opening, the hospitalization time, the follow-up period, and identifiable risk factors of the patients were evaluated.

The patients who were operated due to VVFs and RVFs formed the study groups. The ages of patients, previous surgeries, and diseases were recorded in detail. The patients who had fistula openings greater than 5 mm at the anterior or posterior vaginal walls were not included in the study. The diagnosis of VVF was mainly relied on to tampon test (a gauze was inserted into vagina, bladder was filled with methylene blue dye, staining of gauze with blue confirmed the diagnosis). The size (the longest diameter of the fistula opening was measured including the scar tissue), number, and location of fistulas were noted before the operations. Cystoscopy was performed as an additional procedure in all cases. Bimanual vaginal examination was carried out for the diagnosis of RVF. In addition, the patients with RVF underwent rectoscopy and colonoscopy in order to rule out any malignancy and inflammatory bowel disease. All of the patients were operated by the first author. There were 12 cases with VVF and 15 cases with RVF.

### 2.1. The Surgical Technique for the Repair of VVF

The operation was carried out transvaginally under spinal anesthesia in the dorsal lithotomy position. A single dose of cefotaxime 1 gr was injected preoperatively. A number 8 or 10 French Foley catheter was inserted into the fistula tract. By doing this the fistula tract was determined. The Foley catheter was pulled back so the balloon of the catheter acted as a circular line for circumferentially incising the scar tissue of the fistula tract. After completing the circular incision, the layers of bladder and vaginal wall were dissected off from each other. The bladder submucosa was sutured horizontally with a 4/0 polyglactin suture interruptedly. The bladder musculature (detrusor) was covered over the bladder submucosa with a 4/0 polyglactin sutures continuously. The bladder was filled with methylene blue for seeing any leakage from the suturing side. If any leakage was seen, that part was repaired with additional 4/0 polyglactin sutures interruptedly. The vaginal mucosa was sutured vertically with 2/0 polyglactin interruptedly over the repaired bladder. A Foley catheter was left in bladder for 10 days to provide continuous drainage. Fosfomycin trometamol (Monurol, 3 gr cache) was given as a single dose 1 day after the operation.

### 2.2. The Surgical Technique for the Repair of RVF

The operation was carried out transvaginally under spinal anesthesia in the dorsal lithotomy position. A single dose of cefotaxime 1 gr was injected preoperatively for prophylaxis. A number 8 or 10 French Foley catheter was inserted into the fistula tract. By doing this the fistula tract was determined. The Foley catheter was pulled back so the balloon of the catheter acted as a circular line for circumferentially incising the scar tissue of the fistula tract. After completing the circular incision, the layers of rectum and vaginal mucosa were dissecated off from each other. The rectal submucosa was sutured horizontally with a 4/0 polyglactin suture interruptedly. The rectal serosa was covered over the rectal submucosa with 4/0 polyglactin continuous sutures. The vaginal mucosa was sutured vertically with 2/0 polyglactin interruptedly over the repaired rectum. Ornidazole (Biteral, 500 mg) bid for 5 days and lactulose solution (Duphalac) bid for 2 weeks to all patients after the surgery. All patients were controlled 6 weeks, 6 months, and annually after the operations. The absence of the leakage symptoms were accepted as cure.

## 3. Results

There were 12 cases in the VVF group. The mean age, the mean of the referral time to the hospital, the mean of the longest diameter of the fistula opening at the anterior vaginal wall, the mean of the hospitalization time, and the mean of the follow-up period for this group of the study were 37.8 ± 15.2 years, 14.5 ± 15.5 months, 3.9 ± 0.9 mm, 2.8 ± 0.7 days, and 43 ± 11 months, respectively (Table 1).

All of the VVF openings were ≤5 mm in size and had no trigonal involvement. The anterior vaginal wall length was also normal. The identifiable risk factors for VVF were ascertained and caesarean section was detected as primary risk factor (Table 2).

There were 15 cases in the RVF group. The mean age, the mean of the referral time to the hospital, the mean of the longest diameter of the fistula opening at the posterior vaginal wall, the mean of the hospitalization time, and the mean of the follow-up period for this group of the study were 26.4 ± 9 years, 15.4 ± 42.4 months, 3.2 ± 0.4 mm, 2.8 ± 0.7 days, and 55.2 ± 12 months, respectively (Table 3).

All of the RVF openings were ≤5 mm in size and located between the lower third of the rectum and the lower half of the vagina and above the dentate line. The identifiable risk factors for RVF were also ascertained and prolonged labor was detected as the most important risk factor (Table 4). 

All cases in the both groups were multiparous and the fistula tracts were single. We operated on 11 patients according to VVF and 15 patients according to RVF. All of the cases were primary fistulas. One case, who had VVF after vaginal hysterectomy, applied at 4th week of the operation. In this case, we preferred conservative management because of small fistula size (the longest diameter was 3 mm). Continuous bladder drainage was provided for 8 weeks with a Foley catheter and the fistula tract closed.

The fistula tracts healed completely in 10 out of 11 VVFs and 14 out of 15 RVFs. There was 1 recurrence in each group. The case who had a RVF underwent a second correction transvaginally and 1 year after the second operation no stool or flatus passage was reported. The failure case in the VVF group did not accept a second intervention. No early or late complications have been reported in the rest of the group. The surgical cure rates for VVF and RVF were 91% and 93%, respectively.

## 4. Discussion

Genital fistulas are standing as an important public health problem. However, the underlying pathophysiology can be identified in almost all cases. Ischemic necrosis of the tissues is the main reason of the fistula formation, hence, obstructed prolonged labor due to unattended deliveries, small pelvic dimensions, malpresentation, poor uterine contractions, introital stenosis, gynecologic surgery, radiation therapy, infectious disease such as lymphogranuloma venereum, tuberculosis, syphilis, bladder stones, retained foreign body in the vagina, obstetrical injury, complications of episiotomy, adolescent deliveries, malnutrition, forceps or vacuum applications, Crohn's disease could lead to genital fistulas [4–6]. Our study showed that obstetrical factors have been responsible for the majority of cases. The fistula that developed immediately after cesarean section or vaginal delivery could be managed with continuous bladder drainage for 4–6 weeks in more than 20% of cases [7]. However, there is no consensus on the duration of bladder drainage. After the fistula tract has developed, surgery is the preferred treatment modality. We managed to cure 1 case with continuous bladder drainage for 8 weeks. The surgical success of genitourinary fistulas has been reported to be between 70 and 95% [7–9]. The data is in accordance with our results.

The major reason of RVF is obstetrical trauma. The incidence is 0.1% after vaginal delivery [10]. On the other hand, the incidence of VVF has been quoted as 1/1800 after total abdominal hysterectomy in developed countries [11].

A lot of surgical techniques have been defined for the treatment of fistulas [12, 13]. The choice of treatment varies according to the severity of symptoms, health status of patient, the localization of fistula, etiology, the experience of surgeon, and the existence of sphincteric defect. In our series, we did not prefer transabdominal approach because of the small sizes of fistula openings.

The closure of fistula tract in accordance with the surgical principles, timing of surgery, appropriate anatomical approach, postoperative drainage, the total excision of fistula tract, and adequate tissue flapping increases the success rate. The reasons of postoperative complications are infection, tension on the sutures, local fibrosis due to previous surgery, and ischemia [14]. The scar tissue and adhesions around the fistula could lead to a considerable amount of tissue excision and this could be a risk factor for curing fistula. In our 2 failed surgeries, we thought that this risk played the major role. Conservative treatment should be reserved for the patients with VVF who have good vascularization, no history of radiotherapy, and small fistula tract.

Our study also denotes that RVF is mainly seen in the younger population when compared with VVF. This should be due to higher risk of obstetrical events in younger ages. We should also stress that all the patients in the study group were having primary fistulas. The simple closure technique has been successful for primary repairs; however, the efficiency of the technique in secondary repairs should be evaluated with new studies. Nonetheless, the recurrent cases after simple closure technique could also have another closure surgery unless the fistula opening size >5 mm and have extensive scarring tissue. Another important point is that in our series the number of RVFs was greater than VVFs. This could be due to our inclusion criteria since the patients who had fistula openings greater than 5 mm at the anterior or posterior vaginal walls were not included in the study.

We concluded that, for the primary fistulas either VVF or RVF, if the longest diameter of the openings including the scar tissue is ≤5 mm, the simple closure technique is an efficient surgery.

## Figures and Tables

**Table 1:** The characteristics of vesicovaginal fistula group.

	Mean	Minimum	Maximum	SD*
Age (years)	37.8	22	63	15.2
Referral time (months)	14.5	1	60	15.5
Size (mm)**	3.9	2	5	0.9
Hospitalization time (days)	2.8	1	4	0.7
Followup (months)	43	29	64	11.1

*SD: standard deviation.

**The longest diameter including scar tissue.

**Table 2:** The identifiable risk factors for vesicovaginal fistulas.

Risk factor	Number of cases	Rate (%)
Caesarean section	6	50
Abdominal hysterectomy	5	42
Vaginal hysterectomy	1	8

**Table 3:** The characteristics of rectovaginal fistula group.

	Mean	Minimum	Maximum	SD*
Age (years)	26	21	49	9.0
Referral time (months)	15.4	1	168	42.4
Size (mm)**	3.2	3	5	0.4
Hospitalization time (days)	2.8	2	4	0.7
Followup (months)	55.2	30	64	12.1

*SD: standard deviation.

**The longest diameter including scar tissue.

**Table 4:** The identifiable risk factors for rectovaginal fistulas.

Risk factor	Number of cases	Rate (%)
Prolonged labor	9	60
Vacuum application	1	7
Forceps application	3	20
Abdominal hysterectomy	2	13

## References

[1] Symmonds RE (1984). Incontinence: vesical and urethral fistulas. *Clinical Obstetrics and Gynecology*.

[2] Singh O, Gupta SS, Mathur RK (2010). Urogenital fistulas in women: 5-year experience at a single center. *Urology Journal*.

[3] Rivadeneira DE, Ruffo B, Amrani S, Salinas C (2007). Rectovaginal fistulas: current surgical management. *Clinics in Colon and Rectal Surgery*.

[4] Bazi T (2007). Spontaneous closure of vesicovaginal fistulas after bladder drainage alone: review of the evidence. *International Urogynecology Journal and Pelvic Floor Dysfunction*.

[5] Hilton P, Ward A (1998). Epidemiological and surgical aspects of urogenital fistulae: a review of 25 years’ experience in southeast nigeria. *International Urogynecology Journal and Pelvic Floor Dysfunction*.

[6] Rafique M (2002). Genitourinary fistulas of obstetric origin. *International Urology and Nephrology*.

[7] Waaldijk K (2004). The immediate management of fresh obstetric fistulas. *American Journal of Obstetrics and Gynecology*.

[8] Arrowsmith SD (1994). Genitourinary reconstruction in obstetric fistulas. *Journal of Urology*.

[9] Wall LL, Karshima JA, Kirschner C, Arrowsmith SD, Polan ML (2004). The obstetric vesicovaginal fistula: characteristics of 899 patients from Jos, Nigeria. *American Journal of Obstetrics and Gynecology*.

[10] Venkatesh KS, Ramanujam PS, Larson DM, Haywood MA (1989). Anorectal complications of vaginal delivery. *Diseases of the Colon and Rectum*.

[11] Kumar S, Kekre NS, Gopalakrishnan G (2007). Vesicovaginal fistula: an update. *Indian Journal of Urology*.

[12] Nezhat CH, Nezhat F, Nezhat C, Rottenberg H (1994). Laparoscopic repair of a vesicovaginal fistula: a case report. *Obstetrics and Gynecology*.

[13] Miklos JR, Sobolewski C, Lucente V (1999). Laparoscopic management of recurrent vesicovaginal fistula. *International Urogynecology Journal and Pelvic Floor Dysfunction*.

[14] Patil U, Waterhouse K, Laungani G (1980). Management of 18 difficult vesicovaginal and urethrovaginal fistulas with modified Ingelman-Sundberg and Martuis operations. *Journal of Urology*.

